# Xialla®: a novel medical device for addressing erectile dysfunction associated with veno-occlusive dysfunction

**DOI:** 10.1038/s41443-023-00754-w

**Published:** 2023-08-26

**Authors:** Faysal A. Yafi, Muhammed A. M. Hammad, Dean Elterman

**Affiliations:** 1 Department of Urology, University of California, Irvine, CA USA; 2 Division of Urology, Department of Surgery, University of Toronto, Toronto, ON Canada

**Keywords:** Sexual dysfunction, Cardiovascular diseases

Venous leak or veno-occlusive dysfunction (VOD), a common etiological factor contributing to approximately 75% of erectile dysfunction (ED) cases, has traditionally been a complex condition to manage effectively [1]. Recent advancements have seen the development of the Xialla® (Nigel Shaw, Greater Ottawa Metropolitan Area) constriction ring, a unique, scientifically validated FDA class II wearable device specifically designed to address ED issues related to venous leak.

A clinical trial led by Dr Anthony J. Bella and presented in World Meeting on Sexual Medicine 2016 demonstrated significant enhancement to erectile function in 14 of 21 patients (66%) utilizing the Xialla® device [2]. These findings are further supported by a subsequent trial, which reported satisfactory ED treatment salvage in 6 of 11 men (54%) with VOD [3]. These studies indicate the potential efficacy of the Xialla® device in treating this subset of ED patients with VOD.

Xialla®’s unique design enables application whether the penis is erect or flaccid. In addition, a patented mechanism secures the ring to prevent movement and associated blood leakage during intercourse, potentially improving its therapeutic efficacy compared to traditional rings. Comfort is enhanced with a design that negates the need for a tight fit, typically required by conventional rings.

**Fig. 1:**
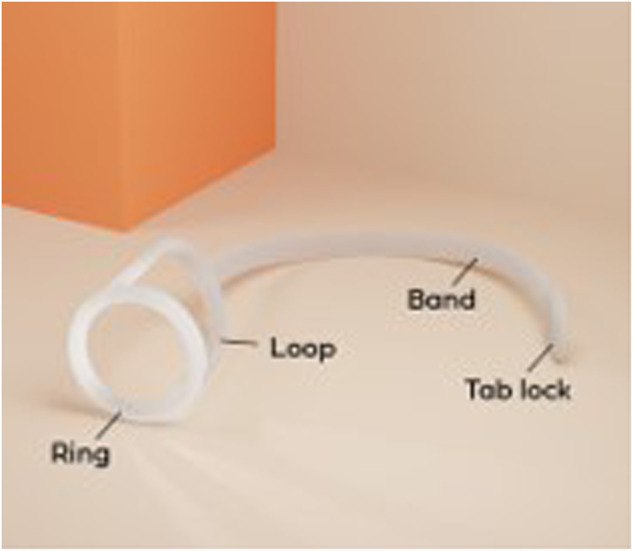
The Xialla device. Identifying the constituents of the device.

**Fig. 2:**
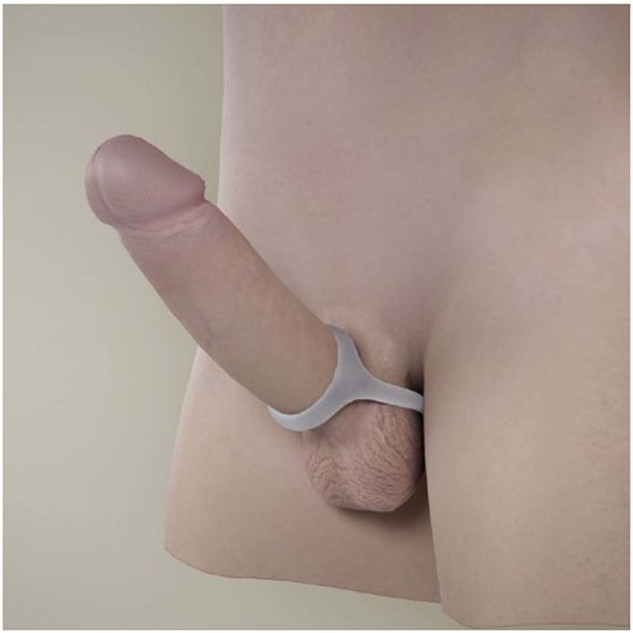
The Xialla device. Placement of the ring and loop.

**Fig. 3:**
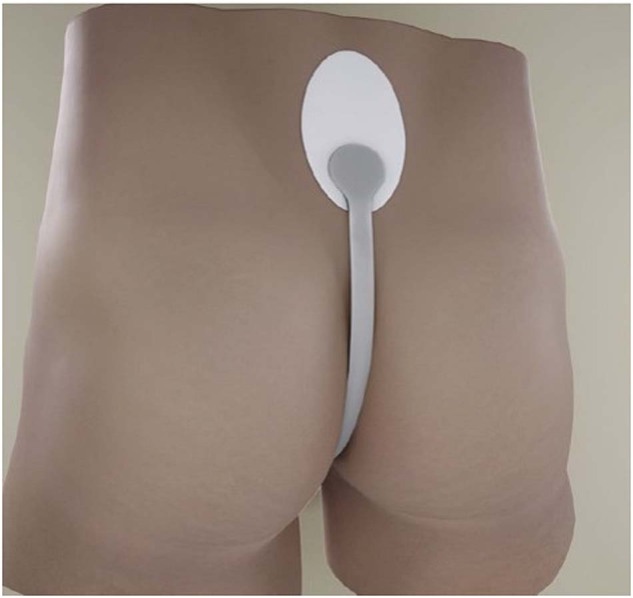
The Xialla device. Placement of the adhesive pad and tab-lock.

The Xialla® device is composed of a soft, stretchy silicone material with a smooth finish, offering abalance of functionality and discretion (Fig. 1). The process of application involves the placement of an adhesive pad on the lower back, followed by the positioning of the ring around the base of the penis. The loop is then extended up and around the scrotum and secured with a tab-lock to the adhesive pad (Figs. 2 and 3).

Beyond its standalone application, Xialla®’s utility extends to concomitant use with other ED therapies, including medications, injections, and pumps. This versatility could potentially make it a beneficial tool in the comprehensive management of ED (particularly VOD), regardless of a patient’s age or medical condition.

Xialla® is a device that has also been evaluated in a clinical trial as a potential treatment option for climacturia (https://xialla.com/blogs/qa/does-xialla-help-with-climacturia). Climacturia refers to the involuntary loss of urine during sexual activity following prostate cancer surgery [4]. The clinical trial, titled “Salvage of Climacturia Non-surgical Treatment Failures using a Patient and Partner-friendly Novel Soft Silicone Occlusion Loop during Sexual Activity,” involved a cohort of five patients who had experienced treatment failure for climacturia and were not interested in surgical correction options. Results from the trial showed promising outcomes, with all five men reporting improvement in climacturia. Four out of the five patients experienced no urine leak associated with sexual activity once the Xialla device was applied. One patient even described significant decreases in climacturia and was able to return to his previous treatment modality with reduced urine loss. While the clinical trial mentioned above demonstrated promising outcomes, it is important to note that it was a small study involving only five patients. Nevertheless, the trial results indicate that Xialla holds potential as a non-surgical option for managing climacturia, providing hope for those experiencing this condition after prostate cancer surgery.

Penile strangulation due to the placement of constricting devices whether metallic or non-metallic is a rare but serious situation that requires urgent attention to avoid vascular compromise and ischemic injury to penile tissues [5]. If the patient forgets to remove the ring before sleeping or leaves it on for extended periods, it can lead to penile ischemia and potentially severe clinical consequences. Management of penile constriction injuries involves the immediate removal of the constricting device to restore blood flow and prevent further tissue damage.

Further research and multicenter trials will be necessary to confirm the potential of this tool and its impact on men affected by ED. The development of the Xialla® device, however, highlights the progressive shift in ED therapeutics, aiming not merely to manage ED symptoms but to address the underlying cause and potentially restore erections to their pre-pathological state. Such advancements signal a promising era in ED therapeutics, with the potential to improve patient outcomes and quality of life.
